# Sleep and Depressive Symptoms in the Morningness/Eveningness-Suicidal Ideation Relationship Depend on School Shift in Mexican Adolescents

**DOI:** 10.3390/jcm10204681

**Published:** 2021-10-13

**Authors:** Arturo Arrona-Palacios, Juan F. Díaz-Morales, Zaida Parra-Robledo, Ana Adan

**Affiliations:** 1 Writing Lab, Institute for the Future of Education, Tecnológico de Monterrey, Monterrey 64849, Mexico; aarronapalacios@bwh.harvard.edu; 2 Division of Sleep and Circadian Disorders, Department of Medicine, Brigham and Women’s Hospital, Boston, MA 02115, USA; 3 Division of Sleep Medicine, Harvard Medical School, Boston, MA 02115, USA; 4 Department of Social Psychology, Work and Individual Differences, Faculty of Psychology, Complutense University of Madrid, 28223 Madrid, Spain; jfdiazmo@ucm.es (J.F.D.-M.); zparra@ucm.es (Z.P.-R.); 5 Department of Clinical Psychology and Psychobiology, School of Psychology, University of Barcelona, 08035 Barcelona, Spain; 6 Institute of Neurosciences, University of Barcelona, 08035 Barcelona, Spain

**Keywords:** sleep, circadian preference, depression, suicidal ideation, adolescence, school shift

## Abstract

The aim was to analyze the morningness/eveningness (M/E) effect on suicidal ideation through sleep and depressive symptoms mediators with school shift (i.e., morning and afternoon) as moderator. In this study, 586 Mexican adolescents, with a mean age of 16.31 ± 0.92, from a public high school in a double-shift school system (298 from the morning shift and 288 from the afternoon shift) participated. Measurements of sleep, morningness/eveningness (circadian preference), depressive symptomology, and suicidal ideation were completed. Adolescents in the afternoon shift reported a later rise time, bedtime, greater time in bed sleep length, and less social jet lag than in the morning shift. Considering the moderated-mediated analysis, circadian preference and suicidal ideation were mediated by both depressive symptoms and school day’s sleep length in the morning shift. In the afternoon shift, no mediation effect was significant. When weekend sleep length was considered in the model, only depressive symptoms had a mediating effect between circadian preference and suicidal ideation in the morning shift; no significant mediating effect was found on the afternoon shift. The results suggest that an afternoon school schedule may act as a protective factor for the adolescent’s mental health and may represent a viable option for future interventions.

## 1. Introduction

Studies have reported that having a short sleep duration on school days and poor sleep quality during adolescence may lead to depression [1,2]. Furthermore, when considering the relationship with morningness/eveningness (M/E), which is the individual preference for activity and sleep during the early morning (morning types) or late at night (evening types), studies in adolescents have found that eveningness is related to greater depressive symptoms [3]. On the other hand, studies about the relationship between suicidal ideation and sleep disturbances have found that shorter sleep duration, severe insomnia, nightmares, and poor sleep quality were associated with more suicidal thoughts [4]. Moreover, when considering M/E, evening types reported greater suicidal thinking than morning types both in clinical and non-clinical samples [5].

Recent clinical reviews about the relationship between sleep duration, depression, and suicidality in adolescents concluded that sleep deprivation significantly predicted higher levels of depressive symptoms and suicidality [6]. Park et al. [7] found that depressive symptoms fully mediated the association between M/E and suicidality. An interesting question would be to analyze whether sleep duration also has a mediating role together with depression in the M/E and suicidal ideation relationship.

This issue becomes highly relevant in the adolescent stage for two reasons. On one hand, because sleep duration is shorter during the school week and longer on the weekend, adolescents’ sleep delay is relatively long compared to children’s and adults’, leading to shorter time in bed during the week due to the early start time of school. Few studies have differentiated both school and weekend sleep duration and its relationship with depression and suicidality. On another hand, most of the studies have been carried out in the morning shift, so it would be very interesting to analyze the M/E-sleep-depression-suicidality relationship in the afternoon shift. During the lifespan, first, there is a shift towards eveningness during the age of puberty, and second, a shift back towards morningness, which could be viewed as a biological marker of the end of adolescence. After reaching adolescence, most people gradually become more and more morning-type. The shift to eveningness occurs around the age of 12–13 years and school start times remain scheduled at early morning hours; this misalignment between biological (evening preference) and social (early school start time) factors has been associated with the greater difficulty of evening types to cope with environmental and social demands. Goldin et al. [8] have suggested a synchrony effect hypothesis, stating that a better alignment between school schedules and the evening type adolescents could be beneficial for their health and performance. Mexican public schools have two different school shifts (i.e., morning and afternoon) and, according to previous studies, students in the afternoon shift have an optimal sleep duration regardless of their circadian preference [9], have better school performance [10], and their overall sleep is not affected while using electronic media before going to bed [11]. Moreover, these studies have illustrated how their circadian preference seems to align better depending on their school shift (i.e., more evening types in the afternoon shift and more morning types in the morning shift). Therefore, we hypothesized that the afternoon shift students’ mental health (depression symptoms and suicidal ideation) will be better compared to those in the morning shift.

The aim of this study is twofold: first to assess the mediating role of sleep and depressive symptoms in the relationship between M/E and suicidal ideation, and second, to assess if the aforementioned mediation model is moderated by morning and afternoon Mexican school shifts.

## 2. Materials and Methods

### 2.1. Participants

The participants were 586 adolescents at a public high school in Reynosa, Tamaulipas (latitude 26°1′00 and longitude 98°14′00) in northeastern Mexico (252 boys and 334 girls). The overall mean age was 16.31 ± 0.92 (mean ± SD) years, and the age range was 15 to 18 years. There were 298 adolescents from the morning shift (school schedule from 07:00 to 13:20), of whom 139 were boys and 159 girls, and 288 adolescents from the afternoon shift (school schedule of 14:00 to 19:30), of whom 113 were boys and 175 girls.

### 2.2. Measures

#### 2.2.1. Sleep Habits

Questions about rising times and bedtimes on weekends and weekdays were adapted from the School Sleep Habits Survey [12]. Several sleep parameters were calculated, based on rise time and bedtime. From these, we calculated time in bed as a proxy to sleep length on school days (School Time in Bed) and weekends (Weekend Time in Bed).

Social jet lag was calculated according to the formula indicated by Wittmann et al. [13] considering the absolute difference between mid-sleep on weekdays (MSW) and mid-sleep on weekends (MSF):

ΔMS = |MSF − MSW|(1)

To obtain mid-sleep, we calculated the middle of time in bed on weekends and weekdays used by Wittmann et al. [13].

#### 2.2.2. Morningness/Eveningness

The Spanish version of the Morningness-Eveningness Scale for Children (MESC) [14,15] was used. The scale is composed of ten items about the preferred timing of certain activities, such as recess, tests, and sleep timing. Scores range from 10 (eveningness) to 43 (morningness). Cut-off points corresponding to the 25th and 75th percentiles of the present sample (22/30 MESC scores) were established to separate evening (*n* = 178), neither (*n* = 247), and morning (*n* = 161) types. Furthermore, the cut-off points separated evening (*n* = 115), neither (*n* = 112), and morning (*n* = 61) types for the afternoon shift and evening (*n* = 63), neither (*n* = 135), and morning (*n* = 100) types for the morning shift. The internal consistency (Cronbach’s alpha) for this sample was α = 0.62.

#### 2.2.3. Suicidal Ideation

The Mexican version of Roberts Suicidal Ideation Scale [16,17] was used, which consists of four items regarding thoughts about death and taking one’s own life over the previous seven days. The score ranges between 0 and 12. The higher the score, the more suicidal ideation, above the cut-off point of 2, people were considered to have suicidal ideation [17]. The internal consistency (Cronbach’s alpha) for this sample was α = 0.85.

#### 2.2.4. Depressive Symptoms

The Mexican version of the short version of CES-D (CESD-7) was used [18]. It follows the same design as the original CES-D [19] (Centre for Epidemiologic Studies Depression Scale), which assesses the severity of depressive symptoms. The overall score ranges between 0 and 21. The higher the score, the more symptoms for depression, and above the cut-off point of 9, people were considered to have clinically significant depressive symptoms [18]. The internal consistency (Cronbach’s alpha) for this sample was α = 0.75.

### 2.3. Procedure

Mexican public high schools follow the same three-year school program scheme as the public secondary school, as described in Arrona-Palacios and Díaz-Morales [10], but instead of considering the full school year, in high school, the school year is divided according to semesters (i.e., a total of 6 semesters). Students are distributed into two classrooms per semester (i.e., 12 classrooms in total). This classroom distribution is used for both school shifts (morning/afternoon). The school shifts are assigned by the school administration according to the results of an admission exam before the adolescents are accepted into high school. Generally, higher scores in the admission exam means adolescents go to the morning shift and lower scores go to the afternoon shift [10]. For this cross-sectional study, the classrooms were randomly selected. A total of 600 questionnaires were distributed, 300 from the morning shift and 300 from the afternoon shift. The application of the questionnaires was during the month of March 2019; the students had approximately 20 min to fill in the questionnaire, which was completed by adolescents in the morning or afternoon shift during their school hours. Data collection was voluntary. Parents and school administrators were informed about the nature and purpose of the study, and written consent to participate was requested.

### 2.4. Statistical Analysis

**Figure 1:**
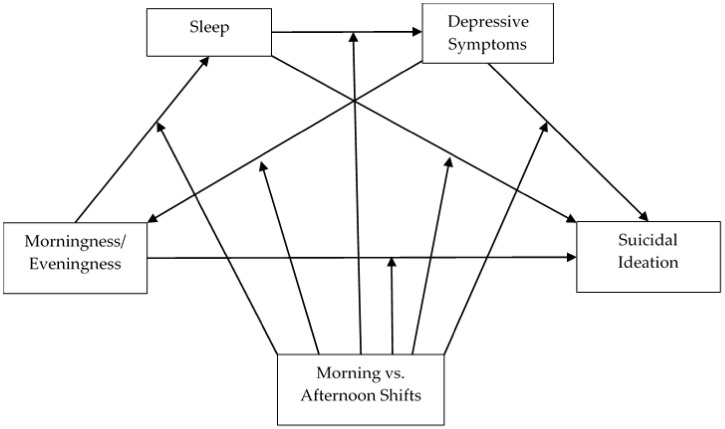
Moderated-Mediated Model to test the morningness/eveningness effect on suicidal ideation through sleep and depressive symptom mediators, with school shift as the moderator.

Multiple analysis of covariance (MANCOVA, age as covariate) was performed to test school shift and sex effects on sleep variables, M/E, suicidal ideation, and depressive symptoms. Partial eta-squared (η_p_^2^) was used as a measure of effect size according to Cohen [20]. To test whether the association between M/E and suicidal ideation was mediated by sleep and depressive symptoms in each school shift, we used the PROCESS macro Model 92 [21] (see Figure 1), testing three moderated-mediation models, differentiated by the measure of sleep: school time in bed, weekend time in bed, and social jet lag.

All analyses were run using the Statistical Package for the Social Sciences program IBM SPSS Statistics 25.0, (Armonk, New York, NY, USA) and PROCESS version 3.5 (http://www.processmacro.org/index.html, accessed on 3 September 2021).

## 3. Results

Considering school shift and morningness/eveningness, significant differences were found (χ^2^ = 27.39, *p* ˂ 0.001). There was a greater percentage of morning types in the morning shift than in the afternoon shift (57.4 vs. 39.9) and in the afternoon shift, there was a greater percentage of evening types than in the morning shift (22.9 vs. 9.1). In addition, considering school shift and depressive symptoms, no significant differences were found (χ^2^ = 1.91, *p* = 0.09), although the percentage with depressive symptoms of morning shift students was greater than those of the afternoon shift (55.4 vs. 49.7). For suicidal ideation, a significant difference was found (χ^2^ = 9.86, *p* ˂ 0.001), morning shift students reported a greater percentage of suicidal ideation than afternoon shift students (36.2 vs. 24.3).

### 3.1. School Shifts, Sex, and Age Effects on Sleep Habits, M/E, Depressive Symptoms, and Suicidal Ideation

A first MANCOVA considered the effects of school shift, sex, and age (covariable) on sleep habits. A preliminary analysis indicated that girls woke up somewhat earlier than boys; for the rest of the sleep variables, there were no significant differences (Table 1 and Table 2).

Considering school shift, on weekdays, students from the morning shift went to bed earlier, woke up earlier, and reported a shorter time in bed than students from the afternoon shift. During the weekend, adolescents from the morning shift went to bed earlier and woke up earlier than those from the afternoon shift. No significant difference was found with time in bed (Table 1 and Table 2). Also, those from the morning shift reported more social jetlag than those from the afternoon shift. Furthermore, a school shift and sex interaction effect on bedtime and rise time was significant during weekday (F_(1,581)_ = 4.54, *p* ˂ 0.01, F_(1,581)_ = 5.18, *p* ˂ 0.05, respectively). Post hoc comparison (Tukey HSD, *p* < 0.001) indicated that the difference in bedtime and rise time between girls and boys was greater in the afternoon shift (00:10 h vs. 00:44 h, 8:33 h vs. 9:05 h, respectively) than in the morning shift (22:59 h vs. 22:58 h, 5:27 h vs. 5:31 h, respectively).

A second MANCOVA considered the effects of school shift, sex, and age (covariable) on M/E, Depressive Symptoms, and Suicidal Ideation. The results indicated that girls reported more symptoms of depression than boys (Table 3 and Table 4). No sex differences were found with M/E and suicidal ideation. Furthermore, students in the morning shift were more morning oriented, and reported more symptoms of depression and more suicidal ideation than afternoon students. Moreover, an interaction effect between school shift and sex with depressive symptoms was significant (Table 4). Post hoc comparison (Tukey HSD, *p* < 0.001) indicated that girls reported more depressive symptoms than boys in the morning shift (10.23 vs. 8.17).

### 3.2. Moderated-Mediation Analysis

To test the morningness/eveningness effect on suicidal ideation through sleep and depressive symptoms mediators, with school shift as the moderator and sex and age as covariables, we tested three moderated-mediation models, changing the measure of sleep as the mediator (school time in bed, weekend time in bed, and social jet lag) in each of them.

The first moderated-mediation model (sleep measured as school time in bed) indicated that, regarding school time in bed (see Table 5), in the morning shift, the more morningness, the more school time in bed (B = 0.04, SE = 0.02, 95% CI (0.003, 0.089), t = 2.10, *p* < 0.05), whereas in the afternoon shift, the more morningness, the less school time in bed (B = −0.04, SE = 0.02, 95% CI (−0.082, 0.001), t = −1.92, *p* < 0.05).

Regarding depressive symptoms, in the morning shift, the more school time in bed, the less depressive symptoms (B = −0.64, SE = 0.18, 95% CI (−0.998, −0.291), t = −3.57, *p* < 0.001), whereas in the afternoon shift, this relationship was weaker (B = −26, SE = 0.12, 95% CI (−0.507, −0.027), t = −2.18, *p* < 0.05).

Considering suicidal ideation, in the morning shift, the greater the presence of depressive symptoms, the greater the suicidal ideation (B = 0.32, SE = 0.03, 95% CI (0.26, 0.39), t = 9.85, *p* < 0.001); this is more apparent than in the afternoon shift (B = 0.21, SE = 0.03, 95% CI (0.14, 0.27), t = 6.83, *p* < 0.001).

The indirect effect of depressive symptoms by school shift was significant (−0.053, 95% CI: (−0.090, −0.019)), indicating that the effect of the M/E on suicidal ideation through depressive symptoms occurs in the morning shift.

Finally, the indirect effect of school time in bed and depressive symptoms by school shift was significant (B = −0.01, SE = 0.005, 95% CI (−0.022, −0.002)), indicating that the effect of M/E on suicidal ideation through school time in bed and the depressive symptoms occurs in the morning shift.

In the second moderated-mediation model, considering the weekend time in bed instead of school time in bed as a mediator (see Table 6), any of the interactions previously described were significant.

Similar to the previous model, the indirect effect of depressive symptoms by school shift was significant (−0.066, 95% CI: (−0.108, −0.031)), indicating that the effect of M/E on suicidal ideation through depressive symptoms occurs in the morning shift.

Finally, in the third moderated-mediation model, considering social jet lag instead of school time in bed and weekend time in bed as mediators (see Table 7), only the indirect effect of depressive symptoms by school shift (−0.066, 95% CI: (−0.108, −0.031)) was significant, indicating that the effect of M/E on suicidal ideation through depressive symptoms occurs in the morning shift.

## 4. Discussion

Previous studies have found that depression mediates the relationship between M/E and suicidality. The first aim of the present study was to examine whether this relationship occurs in the afternoon shift, since most previous studies have been conducted in the morning shift, where evening adolescents are less aligned with the morning social clock imposed by the school schedule. The results of the present study confirm such a mediation effect in the morning shift but not in the afternoon shift, suggesting that, during adolescence, the afternoon shift is beneficial for health.

The second objective was whether the consideration of weekly sleep time, weekend, or social jet lag, together with depression, was implicated in the mediation between M/S and suicidal ideation. The results indicated that only school time in bed together with depression mediated the relationship between M/E and suicidal ideation in the morning shift. Neither weekend time in bed nor social jet lag were significant mediators.

This study has reported for the first time the relevance of the morning and afternoon school shift, as well as school and weekend sleep length, in the morningness-eveningness-depression-suicide relationship. The differences in sleep habits according to school shift were the expected: adolescents from the afternoon shift followed the same trend of getting up late, slept longer, and experienced less social jet lag than adolescents in the morning shift, similar to previous works [9,10,22]. In the morning school schedule, adolescents showed the largest differences (2:40 h) in the sleep time duration between schooldays and weekends, leading to a considerable sleep debt on schooldays, which they compensate for on free days [23]. Furthermore, morning shift students reporting more social jet lag could be an indication that they are experimenting daytime sleepiness [24] and an increased likelihood of having depressive symptoms [25]. Contrary to that, afternoon shift students achieved what the National Sleep Foundation [26] recommends, that adolescents 14–17 years should sleep from 8 to 10 h per day. The data obtained suggest that an afternoon school start time is an effective social environment for a good amount of sleep for adolescents, according to their physiological requirements.

The results indicated that the prevalence of depressive symptoms was similar in adolescents in the morning and the afternoon shift, but suicidal ideation was more prevalent in the morning shift. When considering the mediation analysis, M/E and suicidal ideation were fully mediated by both depressive symptoms and school day’s sleep length in the morning shift. In the afternoon shift, no mediation effect was significant. Furthermore, when weekend sleep length was considered in the mediation model, only depressive symptoms exerted a mediating effect between M/E and suicidal ideation during the morning shift, while there was no significant mediating effect on the afternoon shift.

The majority of the studies on the relationship between sleep duration, depressive symptoms, and suicidal ideation have been carried out considering a morning social environment and found that the more sleep-deprived are the adolescents, the more depressive symptoms or suicidal ideations [3,6]. In our study, the results were similar when considering only the morning shift.

Effectively, depressive symptoms and sleep length both weekdays and weekends were related to suicidal ideation, but the relative contribution of M/E was different according to the school shift. Thus, greater eveningness was associated with suicidal ideation (direct effect) only in the morning shift, and this relationship was partially mediated by depressive symptoms and school days and weekend sleep length (indirect effects). On the other hand, in the afternoon shift, these relationships were not significant. To the extent of our knowledge, this is the first report considering these complex relationships between M/E-depressive symptoms-suicidal ideation by assessing weekdays and weekend sleep duration in morning and afternoon school shifts.

These results add evidence about the negative consequences of misalignment between adolescent internal rhythm and social external cues, such as school timetables, at least in the morning shift. Previous studies conducted with adolescents (mostly in morning shifts) have found that eveningness is a risk factor for health with a higher index of depression [27] and suicidal behavior [28]. In our study, given that the circadian preference of adolescents was in synchrony with the afternoon shift, M/E had no impact on depressive symptoms or suicidal ideation, suggesting that the extreme morning schedule is a risk factor for the health of adolescents, whereas the afternoon schedule is not. Nevertheless, further research is needed.

In earlier investigations, the school start time was considered an important social *zeitgeber* that synchronizes the circadian rhythms of adolescents [29]. However, with the recent body of research on the afternoon school shift [8,9,10,11,22], school start time cannot be considered as a synchronizer, but rather as a social mechanism that will determine the time when adolescents synchronize with the light-dark cycle [30], which is an important physical *zeitgeber* [31]. Interestingly, in the three studies that assess melatonin in school shifts, results have indicated that light-induced melatonin suppression appears first for adolescents in the morning shift, thus synchronizing their circadian rhythms at different times [32,33,34].

One of the hypotheses on the relationships between circadian preference and depressive symptoms is that personality traits, such as neuroticism, could be implicated [27]. Also, severe episodes of depression and high suicidality have been found among evening types, suggesting that personality traits can mediate suicidal behavior [35]. Our results could not point to this hypothesis among adolescents in the afternoon shift, because the direct and indirect effects were not significant.

### 4.1. Implications

There has been a general need for schools around the world to implement later school start times. Delaying school start time may help improve mental health, well-being, healthy weight, and academic performance [36,37]. Moreover, in a study by Peltz et al. [38], when applying a sleep hygiene intervention and considering school start time as a moderator, the direct association was stronger in adolescents with a later school start time, which predicted fewer daily depressive/anxiety symptoms. Our data suggest that extreme delayed school start times such as the ones applied in Mexico can help to prevent insufficient sleep in adolescents. Furthermore, our results reflect that the social schedule may influence whether eveningness can be considered as a risk factor for poor health in adolescents.

### 4.2. Limitations

Several limitations in the current study must be acknowledged. First, this was a cross-sectional study and, according to Sobel [39], mediation analysis in cross-sectional designs only provides partial causality rather than full causality. Second, all the measures used in this study were assessed by self-reports. Future studies may consider using objective measurements (e.g., actigraphy or polysomnography) and conducting clinical interviews to confirm participants’ depressive symptoms and suicidality. Also, measures of daily habits and physical exercise could be included. Third, students in Mexico are selected to their school shifts according to the scores of an admission exam. Non-random selection may cause bias in the results, especially when considering morningness/eveningness assessment, which has been debated in the past [8,22]. Nevertheless, our study, similarly to previous results [10], confirms that random or non-random selection for the school shifts does not alter the findings, because adolescents may have already adapted to their school shift. Future studies could control the season of the year as an important factor that could be proposed in the moderated-mediated model [40]. Finally, future studies on adolescents could test the relevance of biological factors, such as autoimmune diseases, which very often affect the young population themselves as a source of many stress factors, such as reduced activity, failure to fulfil one’s social or scholar roles, and changes in physical appearance [41].

## 5. Conclusions

The main findings suggest that because afternoon shift students may have adjusted or synchronized with the late circadian preference, no direct effect of morningness-eveningness and suicidal ideation was mediated by depressive symptoms and school or weekend sleep length; all this indicates that the afternoon school schedule may act as a protective factor for an adolescent’s mental health and development.

## Figures and Tables

**Table 1:** Means, standard deviation (SD), and the number of participants according to school shift and sex for sleep habits (hh:mm), and social jet lag.

		School Shift						Total		
		Morning			Afternoon					
		Mean	SD	*n*	Mean	SD	*n*	Mean	SD	*n*
School days										
Bedtime	Boys	22:58	01:02	139	00:44	01:38	113	23:46	01:36	252
	Girls	22:59	02:08	159	00:10	01:29	175	23:36	01:55	334
	Total	22:59	01:42	298	00:23	01:34	288	23:40	01:47	586
Rise time	Boys	05:31	00:34	139	09:03	01:41	113	07:09	02:09	252
	Girls	05:27	00:38	159	08:33	01:46	175	07:05	02:03	334
	Total	05:29	00:36	298	08:46	01:45	288	07:06	02:05	586
Time in bed	Boys	6:33	01:12	139	8:20	01:59	113	7:21	01:50	252
	Girls	6:27	02:13	159	8:23	01:53	175	7:28	02:16	334
	Total	6:30	01:49	298	8:22	01:55	288	7:25	02:05	586
Weekends										
Bedtime	Boys	00:22	01:39	139	01:26	01:51	113	00:51	01:49	252
	Girls	00:26	01:37	159	01:00	01:42	175	00:44	01:41	334
	Total	00:24	01:38	298	01:10	01:46	288	00:47	01:45	586
Rise time	Boys	09:26	01:57	139	10:17	02:09	113	09:48	02:05	252
	Girls	09:43	01:46	159	09:57	01:56	175	09:50	01:52	334
	Total	09:35	01:51	298	10:05	02:02	288	09:49	01:57	586
Time in bed	Boys	9:03	01:53	139	8:51	02:20	113	8:57	02:06	252
	Girls	9:16	01:41	159	8:57	01:59	175	9:06	01:51	334
	Total	9:10	01:47	298	8:54	02:07	288	9:02	01:57	586
Social jetlag	Boys	02:39	01:24	139	00:56	01:32	113	01:53	01:41	252
	Girls	02:51	01:49	159	01:06	01:28	175	01:56	01:51	334
	Total	02:45	01:38	298	01:02	01:29	288	01:55	01:47	586

**Table 2:** Statistical data (Fs, *p*-value, and partial eta square) on each sleep variable and social jet lag according to school shift and sex (age as a covariable).

	Age			School Shift			Sex			Shift × Sex		
	F_(1,578)_	*p*-value	η^2^_p_	F_(1,578)_	*p*-value	η^2^_p_	F_(1,578)_	*p*-value	η^2^_p_	F_(1,578)_	*p*-value	η^2^_p_
School days												
Bedtime	0.45	0.500	0.001	107.50	0.001	0.156	4.27	0.039	0.007	4.54	0.033	0.008
Rise time	4.31	0.038	0.007	937.12	0.001	0.617	6.86	0.009	0.012	5.18	0.023	0.009
Time in bed	4.10	0.043	0.007	145.34	0.001	0.200	0.00	0.999	0.000	0.08	0.768	0.001
Weekends												
Bedtime	1.34	0.247	0.002	33.12	0.001	0.054	1.40	0.237	0.002	3.35	0.068	0.006
Rise time	1.19	0.275	0.002	12.30	0.001	0.021	0.01	0.950	0.001	3.96	0.047	0.007
Time in bed	0.01	0.940	0.001	2.33	0.127	0.004	0.93	0.334	0.002	0.144	0.704	0.001
Social Jet lag	0.64	0.422	0.001	160.04	0.001	0.216	2.15	0.143	0.004	0.03	0.857	0.001

**Table 3:** Means, standard deviation (SD), and the number of participants according to school shift and sex for morningness/eveningness, symptoms of depression, and suicidal ideation.

	School Shift									
		Morning			Afternoon			Total		
		Mean	SD	*n*	Mean	SD	*n*	Mean	SD	*n*
Morningness-eveningness	Boys	28.27	4.04	139	26.23	4.28	113	27.35	4.26	252
	Girls	28.48	4.58	159	26.67	4.76	175	27.53	4.76	334
	Total	28.38	4.33	298	26.50	4.58	288	27.45	4.55	586
Depressive symptoms	Boys	8.17	3.79	139	7.93	4.37	113	8.06	4.06	252
	Girls	10.23	4.00	159	8.35	4.15	175	9.24	4.18	334
	Total	9.27	4.03	298	8.18	4.24	288	8.73	4.17	586
Suicidal ideation	Boys	1.54	2.53	139	1.08	2.20	113	1.33	2.40	252
	Girls	1.99	2.96	159	1.16	2.03	175	1.55	2.54	334
	Total	1.78	2.77	298	1.13	2.09	288	1.46	2.48	586

**Table 4:** Statistical data (F, *p*-value, and partial eta square) on morningness/eveningness, symptoms of depression, and suicidal ideation according to school shift and sex (age as a covariable).

	Age			School Shift			Sex			School Shift × Sex		
	F_(1,581)_	*p*-Value	η^2^_p_	F_(1,581)_	*p*-value	η^2^_p_	F_(1,581)_	*p*-Value	η^2^_p_	F_(1,581)_	*p*-Value	η^2^_p_
Morningness/eveningness	7.43	0.01	0.013	20.32	0.001	0.034	1.04	0.31	0.002	0.01	0.91	0.001
Depressive symptoms	8.56	0.001	0.015	13.36	0.001	0.022	12.25	0.00	0.021	4.90	0.03	0.008
Suicidal ideation	5.98	0.01	0.010	12.77	0.001	0.022	1.34	0.25	0.002	0.52	0.47	0.001

**Table 5:** Morningness/eveningness (M/E) effect on suicidal ideation through school time in bed (STB) and depressive symptom (DS) mediators, with school shift as the moderator and sex and age as covariables.

	School Time in Bed		Depressive Symptoms		Suicidal Ideation	
Predictors	*B (SE)*	*t*	*B (SE)*	*t*	*B (SE)*	*t*
Constant	7.85 (1.39)	5.64 ***	10.43 (3.56)	2.93 **	−1.21 (2.02)	−0.60
M/E	0.04 (0.02)	2.10 *	−0.16 (0.05)	−3.08 **	−0.01 (0.03)	−0.55
School Shift	4.38 (0.84)	5.20 ***	−5.18 (2.51)	−2.06 ***	−0.93 (1.55)	−0.60
STB			−0.64 (0.18)	−3.57 ***	−0.22 (0.10)	−2.18 *
DS					0.32 (0.03)	9.85 ***
School Shift × M/E	−0.08 (0.03)	−2.85 **	−0.06 (0.07)	0.83	0.01 (0.04)	0.46
School Shift × STB			0.37 (0.21)	1.98 *	0.17 (0.12)	1.44
School Shift × DS					−0.11 (0.04)	−2.61 **
Sex	−0.06 (0.13)	−0.49	1.2 (0.33)	3.60 ***	−0.15 (0.18)	−0.84
Age	−0.16 (0.07)	−2.24 *	0.43 (0.18)	2.36 *	0.12 (0.10)	1.20
*R^2^*	0.278		0.107		0.27	
*F*	44.69 ***		9.88 ***		44.69 ***	

* *p* < 0.05; ** *p* < 0.01; *** *p* < 0.001.

**Table 6:** Morningness/eveningness (M/E) effect on suicidal ideation through weekend time in bed (WTB) and depressive symptom (DS) mediators, with school shift as moderator and sex and age as covariables.

	Weekend Time in Bed		Depressive Symptoms		Suicidal Ideation	
Predictors	*B (SE)*	*t*	*B (SE)*	*t*	*B (SE)*	*t*
Constant	9.25 (1.68)	5.49 ***	8.30 (3.61)	2.29 *	−2.29 (2.02)	−1.27
M/E	−0.01 (0.02)	−0.15	−0.19 (0.05)	−3.61 ***	−0.02 (0.03)	−0.81
School Shift	−0.52 (1.01)	−0.51	−4.31 (2.57)	−1.67	0.75 (1.55)	0.48
WTB			−0.23 (0.12)	−1.80	−0.02 (0.07)	−0.31
DS					0.34 (0.03)	10.47 ***
School Shift × M/E	−0.01 (0.03)	0.26	0.10 (0.07)	1.42	0.02 (0.04)	0.70
School Shift × WTB			−0.03 (0.17)	−0.18	−0.08 (0.09)	−0.92
School Shift × DS					−0.13 (0.10)	−3.08 **
Sex	0.16 (0.16)	0.97	1.30 (0.33)	3.88 ***	−0.14 (0.18)	−0.75
Age	−0.01 (0.09)	−0.04	0.49 (0.18)	2.68 **	0.13 (10)	1.35
*R^2^*	0.006		0.094		0.253	
*F*	0.70		8.56 ***		21.70 ***	

* *p* < 0.05; ** *p* < 0.01; *** *p* < 0.001.

**Table 7:** Morningness/eveningness (M/E) effect on suicidal ideation through social jet lag (SJL) and depressive symptom (DS) mediators, with school shift as moderator and sex and age as covariables.

	Social Jet Lag		Depressive Symptoms		Suicidal Ideation	
Predictors	*B (SE)*	*t*	*B (SE)*	*t*	*B (SE)*	*t*
Constant	5.69 (1.23)	4.62 ***	6.22 (3.55)	1.75	−2.92 (1.96)	−1.49
M/E	−0.06 (0.01)	−3.42 **	−0.19 (0.05)	−3.50 **	−0.02 (−0.02)	−0.67
School Shift	−3.07 (0.74)	−4.13 ***	−0.01 (0.17)	−1.96 *	0.40 (1.33)	0.30
SJL			−4.39 (2.23)	−0.05	0.04 (0.09)	0.44
DS					0.34 (0.03)	10.55 ***
School Shift × M/E	−0.07	1.83	0.10 (0.07)	1.35	0.02 (0.04)	0.54
School Shift × SJL			−0.06 (0.23)	−27	−0.19 (0.12)	−1.56
School Shift × DS					−0.13 (0.04)	−2.98 **
Sex	−0.15 (0.12)	1.31	1.6 (0.33)	3.74 **	−0.14 (0.18)	−0.79
Age	−0.07 (0.06)	−1.12	0.48 (0.18)	2.65 **	0.12 (0.10)	1.28
*R^2^*	0.267		0.080		0.253	
*F*	42.23 ***		7.14 ***		21.63 ***	

* *p* < 0.05; ** *p* < 0.01; *** *p* < 0.001.

## Data Availability

The data presented in this study are available from the corresponding author upon reasonable request.
